# Suicide rates among patients with first and second primary cancer

**DOI:** 10.1017/S2045796023000690

**Published:** 2023-09-15

**Authors:** Yanting Jiang, Yiqi Wang, Xiaofei Cheng, Ziyang Zhou, Jili Wang, Haogang Yu, Guorong Yao, Zhongjie Lu, Xin Chen, Senxiang Yan, Feng Zhao

**Affiliations:** 1 Department of Radiation Oncology, The First Affiliated Hospital, Zhejiang University School of Medicine, Hangzhou, Zhejiang, P.R. China; 2 Graduate School, Zhejiang University School of Medicine, Hangzhou, Zhejiang, P.R. China; 3 Department of Colorectal Surgery, The First Affiliated Hospital, Zhejiang University School of Medicine, Hangzhou, Zhejiang, P.R. China; 4 Department of Pathology, The First Affiliated Hospital, Zhejiang University School of Medicine, Hangzhou, Zhejiang, P.R. China; 5 Cancer Center, Zhejiang University, Hangzhou, Zhejiang, P.R. China; 6 Institute of Pharmaceutical Biotechnology, Faculty of Medicine, Zhejiang University, Hangzhou, Zhejiang, P.R. China

**Keywords:** psychological burden, second primary cancer, SEER, suicide death

## Abstract

**Aims:**

With advancements in cancer treatments, the survival rates of patients with their first primary cancer (FPC) have increased, resulting in a rise in the number of patients with second primary cancer (SPC). However, there has been no assessment on the incidence of suicide among patients with SPC. This study assessed the occurrence of suicide among patients with SPC and compared them with that in patients with FPC.

**Methods:**

This was a retrospective, population-based cohort study that followed patients with FPC and SPC diagnosed from the National Cancer Institute’s Surveillance, Epidemiology, and End Results (SEER) 17 registries database between 1 January 2000 and 31 December 2019.

**Results:**

For patients with SPC, an age of 85+ years at diagnosis was associated with a higher incidence of suicide death (HR, 1.727; 95% CI, 1.075–2.774), while the suicide death was not considerably different in the chemotherapy group (*P* > 0.05). Female genital system cancers (HR, 3.042; 95% CI, 1.819–6.361) accounted for the highest suicide death among patients with SPC. The suicide death distribution of patients with SPC over time indicated that suicide events mainly occurred within 5 to 15 years of diagnosis. Compared with patients with FPC, patients with SPC in general had a lower risk of suicide, but increased year by year.

**Conclusion:**

The risk of suicide was reduced in patients with SPC compared with patients with FPC, but increased year by year. Therefore, oncologists and related health professionals need to provide continuous psychological support to reduce the incidence of suicide. The highest suicide death was found among patients with female genital system cancer.

## Introduction

Cancer is the leading cause of death worldwide, accounting for more than 10 million deaths each year (Zaimy *et al.*, 2017). The burden of cancer continues to increase, placing a large economic, healthcare and financial burden on society (Fane and Weeraratna, 2020; Lin *et al.*, 2021). Number of new cases of cancer is expected to reach 28.4 million by 2040 (Sung *et al.*, 2021). Thus, identifying the death risk associated with patients having cancer has implications for clinically targeted interventions and treatments.

Suicide has become the 10th leading cause of death in North America (Fazel and Runeson, 2020). First primary cancer (FPC) not only places a huge burden on society but also causes great psychological distress to patients (Dewar *et al.*, 2021; Schofield *et al*., 2003). Statistics show that the incidence of suicide death among patients with cancer was disturbingly high, with 39.72 per 100,000 person-years (Du *et al.*, 2020). Another overall analysis revealed an 85% increased suicide mortality rate among patients with cancer compared with the general population (standard mortality ratio (SMR)  = 1.85, 95% CI = 1.55–2.20) (Heinrich *et al.*, 2022). Besides, cancer treatment and management have changed considerably over the past decades (Carioli *et al.*, 2019; Riley *et al.*, 2019; Vanneman and Dranoff, 2012), contributing to a significant increase in the survival rate among patients with FPC as well as the incidence of second primary cancer (SPC). SPC is not a phenomenon of cancer recurrence or metastasis but rather the occurrence of another cancer distinct from FPC (van der Waal and de Bree, 2010). The standardized incidence risk for any SPC was 1.16 (95% CI 1.12–1.19) (Rombouts *et al.*, 2017). Therefore, more attention should be given to the risk of suicide death among patients with SPC.

Few studies have been conducted on suicide death among patients with SPC. Previous studies among patients with cancer have mainly focused on the suicide death of patients with FPC or a single cancer (Dalela *et al.*, 2016; Kam *et al.*, 2015; Zaorsky *et al.*, 2019), and suicide death has not been assessed in a large sample of patients with SPC. SPC may be a stronger stressor, leading to an increase in suicide death (Yang *et al.*, 2022), or it may account for a decrease in suicide death because of the stronger psychological power acquired from surviving an FPC (Brinkman *et al.*, 2018). As a result, relevant studies concerning suicide death among patients with SPC are urgently needed.

To identify and characterize subgroups of patients with SPC with a higher risk of suicide death, we conducted a population-based cohort study comparing them with patients with FPC. These results are expected to provide a scientific basis for developing specific suicide death management strategies tailored to the needs of patients with SPC, as well as providing insights for the reduction of suicide occurrence among this population.

## Methods

### Data source

Patients with cancer in this retrospective population-based study were screened from the SEER database using SEER*Stat Version 8.4.0.1. The SEER 17 database provides cancer cases from specific geographic areas, covering approximately 26.5% of the U.S. population. The SEER research data include incidence and population data, including patient age, sex, race, year of diagnosis and geographic areas (by SEER registry and county). No ethical approval was necessary as this study was based on a public database (Sturgeon *et al.*, 2019).

### Study population

A total of 5,643,421 patients with cancer were screened from 2000 to 2019 according to the International Classification of Disease Oncology (ICD-O) third edition codes. In our study, FPC was defined as ‘one primary only’, and SPC was defined as ‘1st of 2 or more primaries’ in the SEER database. The inclusion criteria were as follows: (1) cancer types defined by ICD-O third edition codes; (2) pathologically diagnosed; (3) cancer sequence numbers of ‘one primary only’ or ‘1st of 2 or more primaries’; and (4) 6 months after the diagnosis of FPC diagnosis. The exclusion criteria were as follows: (1) unknown survival time and (2) unknown race (Supplemental Figure S1). We conducted separate analyses on system-based cancer sites in the sub-analysis according to the ICD-O third edition codes. The results in current study are presented for patients with cancers of the lymphoma, digestive system, respiratory system, bones and joints, soft tissue including heart, skin excluding basal and squamous, breast, female genital system, male genital system, urinary system, eye and orbit, brain and other nervous system, endocrine system, oral cavity and pharynx, myeloma, leukaemia, mesothelioma, Kaposi sarcoma and miscellaneous.

### Patient variables and outcome assessment

Patient variables were age at diagnosis (years), sex, race, year of diagnosis, socio-demographic factors (i.e., median household income, marital status), tumour characteristics (i.e., stage, cancer site and grade) and cancer treatment (i.e., chemotherapy, radiotherapy and cancer-directed surgery). In the present study, the primary endpoint event was suicide death among patients with FPC and SPC, defined as ‘suicide and self-inflicted injury’ in the SEER database, corresponding with the International Classification of Diseases (ICD) – 10th edition (ICD-10) codes as U03, X60-X84 and Y87.0. Causes of death were determined on the basis of a death certificate recorded and confirmed by the doctor in charge of the patient. The causes of death in the SEER database were categorized according to ICD-10 codes and documented through the National Center for Health Statistics. The follow-up times were from the date of cancer diagnosis to the date of death, loss to follow-up or the last follow-up date (31 December 2019).

### Statistical analysis

Categorical variables at baseline were compared using the chi-square test. As the most popular model, the Cox proportional-hazards regression model is used to examine the predicted values of survival based on covariates such as treatment, age, sex, race and income to predict survival among patients under certain medical conditions. The exponent of the Cox model coefficient provides the instantaneous relative risk of a one unit increase in the relevant covariate. We used univariate Cox proportional hazards (PHs) regression analyses to identify risk factors for suicide death among patients with cancer, and multivariate analyses were employed to examine factors that were significant in univariate analyses (*P* < 0.05) as well as the system-based cancer sites. The PH assumption was also checked. We used Kaplan–Meier (KM) survival curves to show the cumulative incidence of suicide events in patients with cancer with different clinical characteristics. Data analyses were performed using IBM SPSS Statistics (version 21.0.1). All calculations were completed with R software (version 4.1.2). *P* values <0.05 were statistically significant.

## Results

### Study population

A total of 5,643,421 patients with cancer were screened from the SEER database, including 4,931,695 patients with FPC and 711,726 patients with SPC (Table 1). The median follow-up time was 8.32 [interquartile range (IQR) 3.75–13.42] and 12.67 (IQR 7.83–16.58) years for patients with FPC and SPC, respectively. Compared with patients with FPC, patients with SPC were more likely to be male (53.70% versus 50.60%), moderately differentiated (32.20% versus 25.20%), localized stage (61.10% versus 48.40%) and receive cancer-directed surgery (71.10% versus 60.20%). Notably, breast cancer (19.00%) was responsible for the highest number of suicides among patients with SPC, while digestive system cancer (17.70%) had the highest number of suicides among patients with FPC. The suicide death distribution by cancer site and age at diagnosis among FPC and SPC is shown in Supplemental Figure S2. Overall, patients with male genital system cancer had the highest proportion of suicide deaths (>25.00%), followed by digestive system cancers in patients with FPC and SPC of any age group.

**Table 1.** Demographics and clinical characteristics of the study population

	*N* (%)		
Variable	FPC (*N* = 4,931,695)	SPC (*N* = 711,726)	*P*
**Age at Diagnosis (years)**			**<0.001**
≤39	382,340 (7.8)	22,711 (3.2)	
40−49	510,168 (10.3)	58,012 (8.2)	
50−59	1,035,640 (21)	143,173 (20.1)	
60−69	1,338,905 (27.1)	225,627 (31.7)	
70−79	1,061,541 (21.5)	188,390 (26.5)	
80 +	603,101 (12.2)	73,813 (10.4)	
**Sex**			**<0.001**
Female	2,435,701(49.4)	329,200 (46.3)	
Male	2,495,994 (50.6)	382,526 (53.7)	
**Race**			**<0.001**
White	4,019,939 (81.5)	604,556 (84.9)	
Black	517,236 (10.5)	63,283 (8.9)	
Others^a^	394,520 (8.0)	43,887 (6.2)	
**Marital Status**			**<0.001**
Married	2,705,497 (54.9)	420,508 (59.1)	
Unmarried	1,857,424 (37.7)	232,989 (32.7)	
Unknown	368,774 (7.5)	58,229 (8.2)	
**Median Household Income** (US$)			**<0.001**
<40,000	181,590 (3.7)	23,243 (3.3)	
40,000—49,999	476,478 (9.7)	64,918 (9.1)	
50,000—59,999	736,562 (14.9)	103,197 (14.5)	
60,000—69,999	1,519,407 (30.8)	210,981(29.6)	
>70,000	2,016,612 (40.9)	309,279 (43.5)	
Unknown	1046 (0)	108 (0)	
**Year of Diagnosis**			**<0.001**
2000−2004	1,073,193 (21.8)	225,912 (31.7)	
2005−2009	1,173,232 (23.8)	210,028 (29.5)	
2010−2014	1,266,695 (25.7)	168,177 (23.6)	
2015−2019	1,418,575 (28.8)	107,609 (15.1)	
**Grade**			**<0.001**
Well differentiated	432,268 (8.8)	75,825(10.7)	
Moderately differentiated	1,242,503 (25.2)	229,526 (32.2)	
Poorly differentiated	974,598 (19.8)	142,221 (20)	
Undifferentiated	171,325 (3.5)	24,563 (3.5)	
Other/Unknown	2,111,001 (42.8)	239,591 (33.7)	
**Stage**			**<0.001**
Localized	2,387,569 (48.4)	434,602 (61.1)	
Regional	1,082,650 (22)	154,963 (21.8)	
Distant	1,140,152 (23.1)	86,134 (12.1)	
Unknown	321,324 (6.5)	36,027 (5.1)	
**Cancer-directed Surgery**			**<0.001**
Performed	2,969,067(60.2)	506,069 (71.1)	
Not performed	1,922,584 (39)	200,856 (28.2)	
(continued)			
Unknown	40,044 (0.8)	4,801 (0.7)	
**Radiotherapy**			**<0.001**
Yes	1,469,326 (29.8)	204,210 (28.7)	
No/Unknown	3,462,369 (70.2)	507,516 (71.3)	
**Chemotherapy**			**<0.001**
Yes	1,572,875 (31.9)	181,321 (25.5)	
No/Unknown	3,358,820 (68.1)	530,405 (74.5)	
**Cancer Site**			**<0.001**
Lymphoma	245,321 (5)	34,864(4.9)	
Digestive system	872,513 (17.7)	104,036 (14.6)	
Respiratory system	536,089 (10.9)	53,331 (7.5)	
Bones and joints	11,682 (0.2)	886 (0.1)	
Soft tissue including heart	38,418 (0.8)	4,329 (0.6)	
Skin excluding basal and squamous	229,018 (4.6)	56,227 (7.9)	
Breast	786,950 (16)	135,552 (19)	
Female genital system	323,341 (6.6)	38,873 (5.5)	
Male genital system	832,555 (16.9)	124,329 (17.5)	
Urinary system	339,913 (6.9)	78,679 (11.1)	
Eye and orbit	6,043 (0.1)	893 (0.1)	
Brain and other nervous system	77,196 (1.6)	2,694 (0.4)	
Endocrine system	162,815 (3.3)	16,244 (2.3)	
Oral cavity and pharynx	118,585 (2.4)	22,951 (3.2)	
Myeloma	69,683 (1.4)	6,712 (0.9)	
Leukaemia	141,644 (2.9)	15,270 (2.1)	
Mesothelioma	9,970 (0.2)	385 (0.1)	
Kaposi Sarcoma	6,021 (0.1)	840 (0.1)	
Miscellaneous	123,938 (2.5)	14,631 (2.1)	

FPC = first primary cancer, SPC = second primary cancer.

a includes American Indian/Alaska Native and Asian/Pacific Islander.

### Cumulative incidence of suicide death among patients with cancer with KM analysis

**Figure 1.**
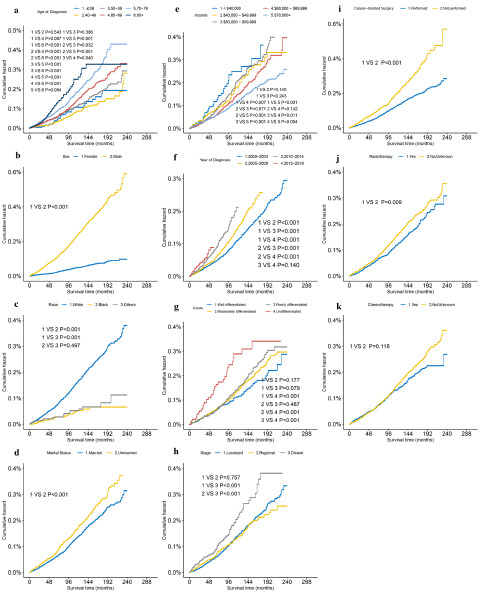
Kaplan–Meier survival analysis of patients with second primary cancer.

**Figure 2.**
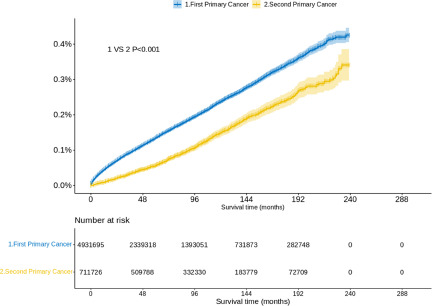
Probability of suicide among patients with first and second primary cancer.

The number of suicide death for FPC and SPC were 6801 and 851, respectively. When considering death by suicide as the outcome, the 5-year survival probability for patients with FPC and SPC was found to be 99.86% and 99.94%, respectively. We further analysed the cumulative incidence of suicide death with the KM test. Figure 1 presents the results for suicide deaths in patients with SPC. Overall, the risk of suicide increased year by year in patients with SPC. Older age at diagnosis was associated with a higher incidence of suicide death among patients with SPC (*P* < 0.001) (Fig. 1a) and the suicide death was not significantly different for patients undergoing chemotherapy (*P* = 0.118) (Fig. 1k). The year of diagnosis was not statistically significant for the risk of suicide among patients with FPC (*P* > 0.05) (Supplementary Figure S3). In addition, we observed a significantly lower suicide death for patients with SPC than for patients with FPC (*P* < 0.001) (Fig. 2).

### Cox PHs regression analysis among patients with FPC and SPC

**Figure 3.**
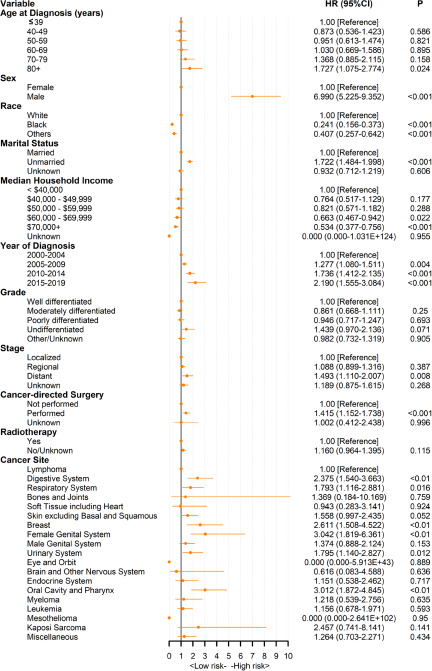
Multivariate Cox proportional hazards regression analyses of patients with second primary cancer. Lymphoma is used as the reference for Cox proportional hazard model.

We used a Cox PHs regression model to estimate risk factors for survival time. In the univariate Cox analysis, older age, male sex, white race, unmarried status, advanced stage, poorer differentiation and untreated (including cancer-related surgery and radiotherapy) patients had a higher risk of suicide in both the FPC and SPC populations. Notably, chemotherapy was statistically significant for patients with FPC (*p* < 0.001) but not for patients with SPC (*P* = 0.118) in the univariate Cox analysis (Supplemental Tables S1 and S2). Multivariate Cox PHs regression further included cancer sites and statistically significant factors in univariate Cox analysis (*P* < 0.05). Figure 3 depicts a forest plot of multivariate Cox PHs regression analysis of patients with SPC. The results were basically consistent with single factors. Notably, cancer grade (*P* > 0.05), as well as radiotherapy (*P* = 0.115), were not statistically significant for suicide death in patients with SPC. Patients aged ≥80 years had a higher suicide death than patients with cancer aged ≤39 years (HR, 1.73; 95% CI, 1.08–2.77). Compared with patients with lymphoma, patients with female genital system cancer (HR, 3.04; 95% CI, 1.82–6.36) had the highest risk of suicide. In addition, there was no significant difference in suicide death among patients with different malignancies of the female genital system, including cervix uteri, uterus, ovary, vagina and vulva (Supplemental Table S3). Among patients with FPC, patients with mesothelioma (HR, 3.17; 95% CI, 2.02–4.98) had the highest suicide death, followed by patients with oral cavity and pharynx cancer (HR, 2.63; 95% CI, 2.24–3.09) (Supplemental Figure S4).

### Suicide distribution of cancer site and survival time

**Figure 4.**
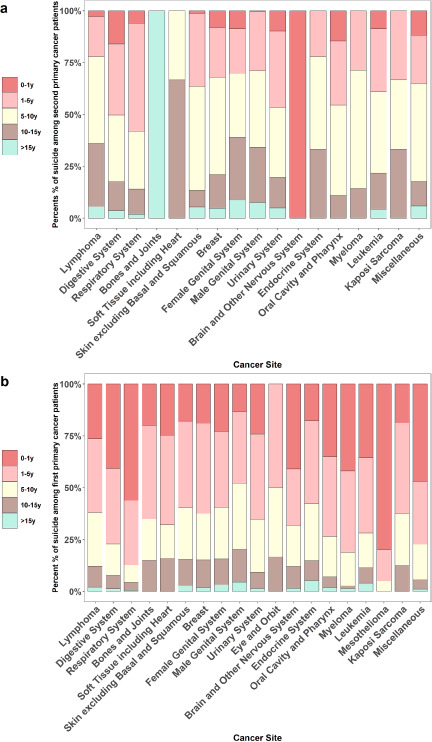
Follow-up trends of patients with first and second primary cancer from cancer diagnosis to suicide.

In the suicide death distribution of patients with SPC, suicide events occur mainly within 5 to 15 years after diagnosis. Respiratory system cancer had the highest suicide proportion within 5 years (Fig. 4a). Strikingly, patients with FPC accounted for a significantly higher proportion of suicides within the first year and within 5 years, and more than 50.00% of patients presented with suicidal behaviour during 5 years of follow-up (Fig. 4b).

## Discussion

This is a large-scale study exploring suicide death among patients with SPC and FPC based on U.S. registry data. After adjusting for several potential confounders, we observed a reduced risk of suicide death among patients with SPC compared to patients with FPC throughout the follow-up period. However, patients with SPC had an increasing risk of suicide as the diagnosis year increased. Our study explored the risk of suicide in patients with SPC by comparison with patients with FPC, providing a scientific basis for suicide death management strategies for patients with SPC.

Previous studies have mainly focused on suicide by individual cancer type (Chen *et al.*, 2021a; 2021b), or in patients with FPC (Chen *et al.*, 2022; Ma *et al.*, 2021), which has limited our knowledge of the suicide death among patients with SPC with extensive cancer types. Our study found a reduction in suicide death with an SPC diagnosis compared to an FPC diagnosis. Although there is a lack of similar research, our study provides direction for future exploration. Previous related studies differ in part from our study. Yang et al. reported a higher risk of suicide among patients with SPC compared with patients with FPC (Yang *et al.*, 2022). This disparity could be attributed to the inconsistency in the year of diagnosis within their sample. Their study included data from 1975 to 2016, while our study covered the period from 2000 to 2019. Notably, during earlier times, patients with SPC had poor access to good care, which likely contributed to an higher risk of death by suicide. Consequently, the overall suicide death reported by Yang et al. for patients with SPC would be likely influenced by the poor access to good care before 2000. Additionally, the survival time of patients from SPC diagnosis to suicide increased significantly, which may be associated with greater psychological power and adaptation to cancer-related treatment (Schofield *et al*., 2003). Samson and Zerter pointed out that the experience of cancer is an experience that promotes great personal and spiritual growth, which can provoke personal growth and transformation (Samson and Zerter, 2003).

Notably, the risk of suicide increased year by year in patients with SPC, despite the reduction in the risk of suicide and the increase in survival time from diagnosis to suicide. Of note, we found that patients with FPC have a decreased risk of suicide as the year of diagnosis increases, while it increases among patients with SPC. Our results are in line with previous studies. For example, Ma, Wen et al. reported a significant decrease in age-adjusted suicide death from 1975 to 2017 in almost all patients with solid tumours (Ma *et al.*, 2021). Improvements in living standards and medical technology may help reduce suicide death in patients with FPC (D’Anci *et al.*, 2019) . For patients with SPC, the diagnosis of SPC is devastating and causes great psychological stress (Kazak *et al.*, 2010; Mitchell *et al.*, 2011). At the same time, the treatment modality that patients receive after the treatment of FPC may have an effect on the occurrence of SPC (Chaturvedi *et al.*, 2007; El-Gamal and Bennett, 2006), which may lead to patients’ disappointment with the treatment modality (Schmid *et al*., 2005), causing higher suicide death year by year. Although the overall risk of suicide decreased over the year of diagnosis for patients with SPC compared with those with FPC, patients with SPC have an increased risk of suicide as the year of diagnosis increases, underscoring the importance of continuous suicide interventions.

Similar to the results of previous FPC studies (Misono *et al.*, 2008; Saad *et al.*, 2019; Yang *et al.*, 2021), our study found characteristics associated with high suicide death among patients with SPC. Older patients (85+) had a higher risk of suicide, which may be related to poorer health status, low resistance, low quality of life, loneliness and depression among older patients with SPC (Kam *et al.*, 2015; Gaitanidis *et al.*, 2018; Yang *et al.*, 2019). However, it is worth noting that regardless of cancer, older people, particularly those over 80 years of age, are at higher risk of suicide, especially when facing conditions such as chronic pain, dependence on others, loneliness, feelings of abandonment and loss of meaning. The goal of SPC suicide prevention may be included in a broader one to improve the quality of life for elder and eliminate factors that contribute to depression (Leo, 2022). In addition, the suicide death is markedly higher among men with SPC than women, which may be due to influence by conventional masculine social norms and the worse emotion regulating than women (Möller-Leimkühler, 2002; Nolen-Hoeksema, 2012). White patients were more likely to commit suicide than patients of other races, which may be attributable to the majority of study subjects being white (84.9%) and to the religious beliefs of various ethnic groups (Yu *et al.*, 2022). Additionally, the marital status of patients with SPC was also linked to suicide death. We found that unmarried patients with SPC had a higher risk of suicide. Studies have revealed that married patients could receive more care and socioeconomic support from their partners, who are less likely to be depressed and commit suicide (Aizer *et al.*, 2013). Our study also found that patients with SPC with a median household income of over $60,000 have a lower risk of suicide. This finding corresponded to another population-based study (Suk *et al.*, 2021), which may be due to the lack of mental healthcare for patients with cancer living in low-income areas.

Intriguingly, cancer grade, chemotherapy and radiotherapy had no significant effect on suicide death among patients with SPC. This may be due to the tolerance of cancer and related treatments and psychological preparation after FPC treatment. This result indicates that radiotherapy and chemotherapy can be recommended more positively in treating patients with SPC. Strikingly, patients with female genital system cancer harboured the highest suicide death among patients with SPC in the current study. The sexual impact of gynaecologic cancer treatment on physical aspects is well documented (Abbott-Anderson and Kwekkeboom, 2012; Audette and Waterman, 2010; Boa and Grénman, 2018). Ward et al. reported that patients with gynaecologic malignancies had a 1.3 times higher incidence of suicide than patients with other cancer types (Ward *et al.*, 2013). The high risk of suicide in patients with female genital system cancer may be multifactorial. Similar to patients with other cancers, patients with female genital system cancer bear a significant psychological burden after a cancer diagnosis (Boa and Grénman, 2018; Pignata *et al.*, 2001). In addition, previous studies have shown that 50% of women with female genital system cancer may experience anatomical changes in their genitalia while undergoing treatment, which may have an irreversible impact on sexual function and may lead to changes in relationships with sexual partners, resulting in anxiety and depression (Andersen and van Der Does, 1994; Stead *et al.*, 2007). Furthermore, several previous studies have shown that patients with female genital system cancer express concerns related to negative body image (Carmack Taylor *et al.*, 2004). Above all, the combination of these emotional and psychological stressors may increase the risk of suicide death in women with genital system cancer.

These results further elucidate the psychological stress associated with a diagnosis of SPC and call for attention to the mental health status of patients with SPC, particularly those diagnosed in recent years who have female genital system cancer, are 85+ years of age, are white and are unmarried. Those patients need psychological interventions such as effective psychosocial care and effective suicide death screening. Given the increased risk of suicide in patients with SPC over the years, continued psychological support is of greater significance than early psychological support for these priority patients.

The major strength of our study is the large population sample of patients with SPC and FPC in the United States. The large sample size enables us to perform detailed analyses of all subgroups, increasing the reliability of our results. Using ample information on cancer characteristics and treatment modality, we are able to analyse the factors related to suicide in patients with cancer more deeply in our analysis.

Our study has some limitations. First, the specific cause of death is often difficult to identify, and the ICD-10 cause of death code is unique, so there may be a possibility of misclassification bias for death causes in the SEER database (Horn *et al.*, 2020; Zaorsky *et al.*, 2017). Second, the comorbid medical and psychiatric conditions were not assessed, including factors that could influence the incidence of cancer, such as alcohol and tobacco use, which may be associated with their own risk of suicide. Likewise, supportive psychosocial care was not assessed, although combining cancer treatments with supportive care can improve prognosis while reducing adverse effects like suicide death. Third, we are unable to affirm whether the suicide event is secondary to the diagnosis of cancer, other diseases or dramatic changes in life circumstances that occurred in the interval after the cancer diagnosis. Fourth, it is difficult to estimate the effect of time-varying trends, such as therapeutic advances and people’s perceptions of cancer over the past 20 years (de Vries *et al.*, 2021). Finally, caution should be exercised when generalizing the findings to other countries, as the SEER 17 database provides data from specific geographic area.

## Conclusions

Compared with patients with FPC, patients with SPC in general had a lower risk of suicide. However, patients with SPC have an increasing risk of suicide as the year of diagnosis increases. Therefore, oncologists and related health professionals need to provide continuous psychological support to reduce the incidence of suicide, especially for those diagnosed in the most recent year, age of 85+ years, white race and unmarried patients. The results of the current study may provide some scientific basis for the development of a comprehensive suicide death scoring system for screening suicide death in patients with SPC. Larger and longer follow-up cohort studies are needed to validate the SPC–suicide correlation in the future.

## Supporting information

Jiang et al. supplementary material Jiang et al. supplementary material

## Data Availability

The datasets are publicly available from the SEER database (http://seer.cancer.gov).

## Supplementary material

The supplementary material for this article can be found at https://doi.org/10.1017/S2045796023000690.
