# Supplementary material for: Suicide rates among patients with first and second primary cancer
Source: Epidemiol Psychiatr Sci. 2023 Sep 15;32:e57. doi: 10.1017/S2045796023000690 (PMC10539740; doi:10.1017/S2045796023000690)
Supplement: Jiang et al. supplementary material [file S2045796023000690sup001.docx]

**List of Supplemental Materials**

**Supplementary Table 1. Univariate Cox proportional hazards regression analyses of patients with second primary cancer (P2)**

**Supplementary Table 2. Univariate Cox proportional hazards regression analyses of patients with first primary cancer (P4)**

**Supplementary Table 3. Cox proportional hazards regression analyses of second primary cancer patients with female genital system (P6)**

**Supplementary Figure 1. Study population flow chart (P7)**

**Supplementary Figure 2. The proportion of deaths among second and first primary cancer patients (P8)**

**Supplementary Figure 3. Kaplan-Meier survival analysis of patients with first primary cancer (P9)**

**Supplementary Figure 4. Multivariate Cox proportional hazards regression analyses of patients with first primary cancer (P10)**

**Supplementary Table 1. Univariate analysis of clinical variables associated with suicide death among second primary cancer patients**

| **Variable** | **HR (95%CI)** | | ***P*** |
| --- | --- | --- | --- |
| **Age at Diagnosis (years)** |  | |  |
| ≤39 | | 1.00 [Reference] |  |
| 40-49 | | 0.861 (0.532-1.395) | 0.543 |
| 50-59 | | 1.208 (0.788-1.853) | 0.386 |
| 60-69 | | 1.478 (0.974-2.244) | 0.067 |
| 70-79 | | 2.000 (1.315-3.043) | 0.001 |
| 80+ | | 2.546 (1.607-4.035) | <0.001 |
| **Sex** | |  |  |
| Female | | 1.00 [Reference] |  |
| Male | | 5.108 (4.240-6.153) | <0.001 |
| **Race** | |  |  |
| White | | 1.00 [Reference] |  |
| Black | | 0.274 (0.178-0.423) | <0.001 |
| Others | | 0.340 (0.216-0.536) | <0.001 |
| **Marital Status** | |  |  |
| Married | | 1.00 [Reference] |  |
| Unmarried | | 1.279 (1.106-1.478) | <0.001 |
| Unknown | | 1.042 (0.803-1.352) | 0.758 |
| **Median Household Income** | |  |  |
| < $40,000 | | 1.00 [Reference] |  |
| $40,000 - $49,999 | | 0.746 (0.505-1.101) | 0.140 |
| $50,000 - $59,999 | | 0.805 (0.560-1.158) | 0.243 |
| $60,000 - $69,999 | | 0.621 (0.438-0.880) | 0.007 |
| $70,000+ | | 0.488 (0.346-0.689) | <0.001 |
| **Year of Diagnosis** | |  |  |
| 2000-2004 | | 1.00 [Reference] |  |
| 2005-2009 | | 1.353 (1.146-1.596) | <0.001 |
| 2010-2014 | | 1.941 (1.586-2.374) | <0.001 |
| 2015-2019 | | 2.501 (1.785-3.506) | <0.001 |
| **Grade** |  | |  |
| Well differentiated | | 1.00 [Reference] |  |
| Moderately differentiated | | 1.184 (0.927-1.512) | 0.177 |
| Poorly differentiated | | 1.265 (0.973-1.645) | 0.079 |
| Undifferentiated | | 2.287 (1.572-3.328) | <0.001 |
| Other/Unknown | | 1.287 (1.005-1.650) | 0.046 |
| **Stage** | |  |  |
| Localized | | 1.00 [Reference] |  |
| Regional | | 0.973 (0.816-1.159) | 0.757 |
| Distant | | 1.566 (1.254-1.956) | <0.001 |
| Unknown | | 1.326 (1.004-1.749) | 0.046 |
| **Cancer-directed Surgery** | |  |  |
| Performed | | 1.00 [Reference] |  |
| Not performed | | 1.709 (1.482-1.970) | <0.001 |
| Unknown | | 1.240 (0.514-2.991) | 0.632 |
| **Radiotherapy** | |  |  |
| Yes | | 1.00 [Reference] |  |
| No/Unknown | | 1.227 (1.053-1.428) | 0.009 |
| (continued)  **Chemotherapy** | |  |  |
| Yes | | 1.00 [Reference] |  |
| No/Unknown | | 1.141 (0.967-1.347) | 0.118 |

^a^ includes American Indian/Alaska Native and Asian/Pacific Islander.

**Supplementary Table 2. Univariate analysis of clinical variables associated with suicide death among first primary cancer patients**

| **Variable** | **HR (95%CI)** | | ***P*** |
| --- | --- | --- | --- |
| **Age at Diagnosis (years)** |  | |  |
| ≤39 | | 1.00 [Reference] |  |
| 40-49 | | 1.356 (1.203-1.529) | <0.001 |
| 50-59 | | 1.471 (1.320-1.640) | <0.001 |
| 60-69 | | 1.705 (1.534-1.895) | <0.001 |
| 70-79 | | 2.146 (1.927-2.389) | <0.001 |
| 80+ | | 2.243 (1.984-2.535) | <0.001 |
| **Sex** | |  |  |
| Female | | 1.00 [Reference] |  |
| Male | | 4.887 (4.589-5.205) | <0.001 |
| **Race** | |  |  |
| White | | 1.00 [Reference] |  |
| Black | | 0.319 (0.280-0.364) | <0.001 |
| Others ^a^ | | 0.599 (0.538-0.668) | <0.001 |
| **Marital Status** | |  |  |
| Married | | 1.00 [Reference] |  |
| Unmarried | | 1.392 (1.323-1.464) | <0.001 |
| Unknown | | 1.540 (1.418-1.673) | <0.001 |
| **Median Household Income** | |  |  |
| < $40,000 | | 1.00 [Reference] |  |
| $40,000 - $49,999 | | 1.052 (0.916-1.209) | 0.475 |
| $50,000 - $59,999 | | 0.899 (0.787-1.028) | 0.121 |
| $60,000 - $69,999 | | 0.779 (0.685-0.885) | <0.001 |
| $70,000+ | | 0.685 (0.604-0.778) | <0.001 |
| Unknown | | 1.473 (0.472-4.594) | 0.505 |
| **Year of Diagnosis** | |  |  |
| 2000-2004 | | 1.00 [Reference] |  |
| 2005-2009 | | 0.980 (0.921-1.042) | 0.521 |
| 2010-2014 | | 0.958 (0.895-1.025) | 0.215 |
| 2015-2019 | | 0.908 (0.833-0.989) | 0.028 |
| **Grade** |  | |  |
| Well differentiated | | 1.00 [Reference] |  |
| Moderately differentiated | | 1.547 (1.400-1.709) | <0.001 |
| Poorly differentiated | | 1.874 (1.692-2.076) | <0.001 |
| Undifferentiated | | 2.076 (1.770-2.434) | <0.001 |
| Other/Unknown | | 1.698 (1.539-1.875) | <0.001 |
| **Stage** | |  |  |
| Localized | | 1.00 [Reference] |  |
| Regional | | 1.119 (1.053-1.189) | <0.001 |
| Distant | | 1.741 (1.629-1.861) | <0.001 |
| Unknown | | 1.672 (1.527-1.832) | <0.001 |
| (continued) | |  |  |
| **Cancer-directed Surgery** | |  |  |
| Performed | | 1.00 [Reference] |  |
| Not performed | | 2.020 (1.925-2.121) | <0.001 |
| Unknown | | 2.259 (1.810-2.819) | <0.001 |
| **Radiotherapy** | |  |  |
| Yes | | 1.00 [Reference] |  |
| No/Unknown | | 1.194 (1.132-1.259) | <0.001 |
| **Chemotherapy** | |  |  |
| Yes | | 1.00 [Reference] |  |
| No/Unknown | | 1.168 (1.105-1.234) | <0.001 |

^a^ includes American Indian/Alaska Native and Asian/Pacific Islander.

**Supplementary Table 3. Cox proportional hazards regression analyses of second primary cancer patients with female genital system**

| **Variable** | **HR (95%CI)** | | ***P*** |
| --- | --- | --- | --- |
| **Female Genital System** |  | |  |
| Cervix Uteri | | 1.00 [Reference] |  |
| Uterus | | 0.477 (0.056-4.093) | 0.5 |
| Ovary | | 0.256 (0.033-1.985) | 0.192 |
| Vagina | | 0.405 (0.047-3.466) | 0.409 |
| Vulva | | 0.000 (0.000-1.321E+265) | 0.972 |
| Unknown | | 0.202 (0.013-3.238) | 0.259 |

**Supplementary Figure 1. Study population flow chart**


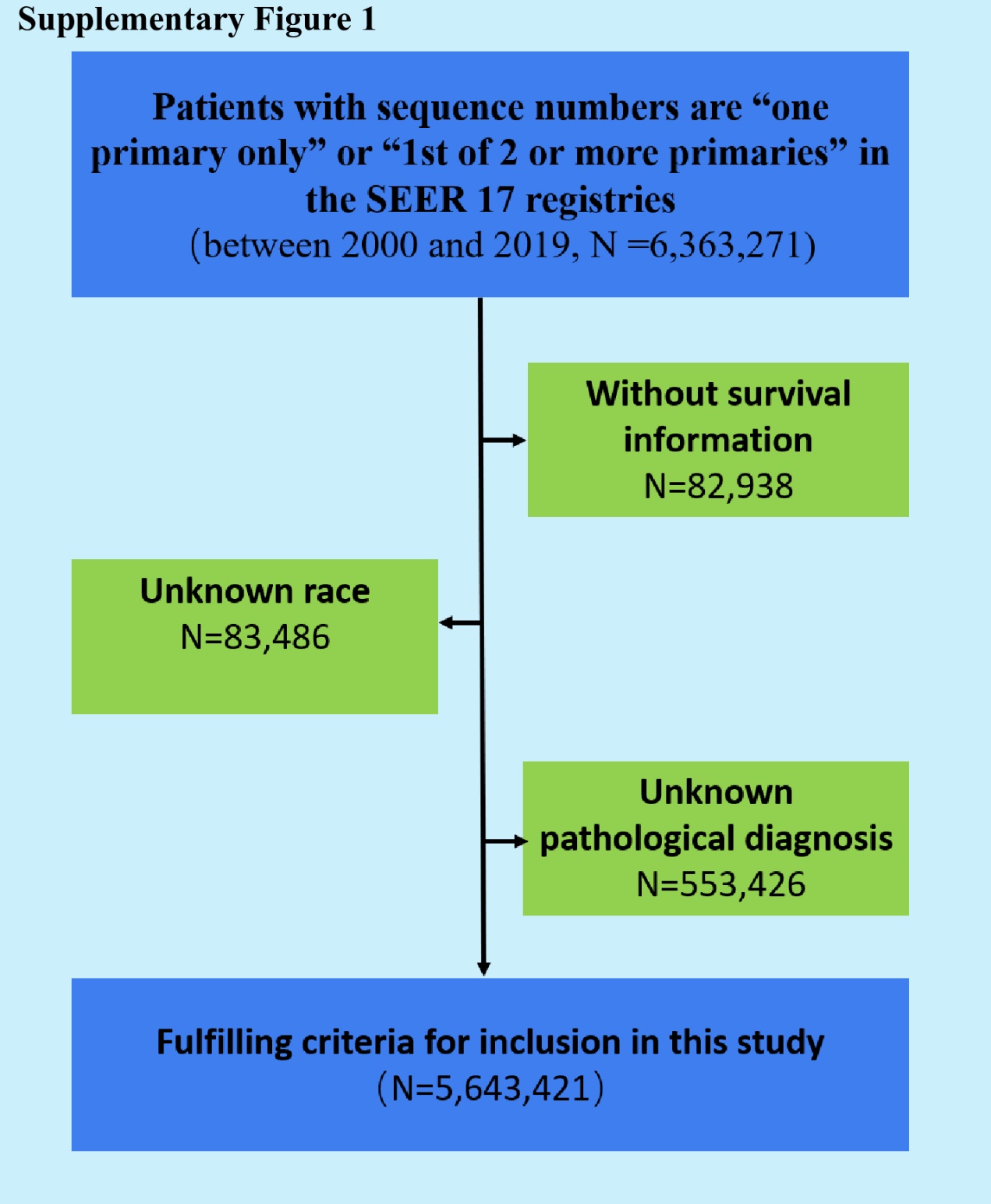


**Supplementary Figure 2.** The proportion of deaths among second and first primary cancer patients

**
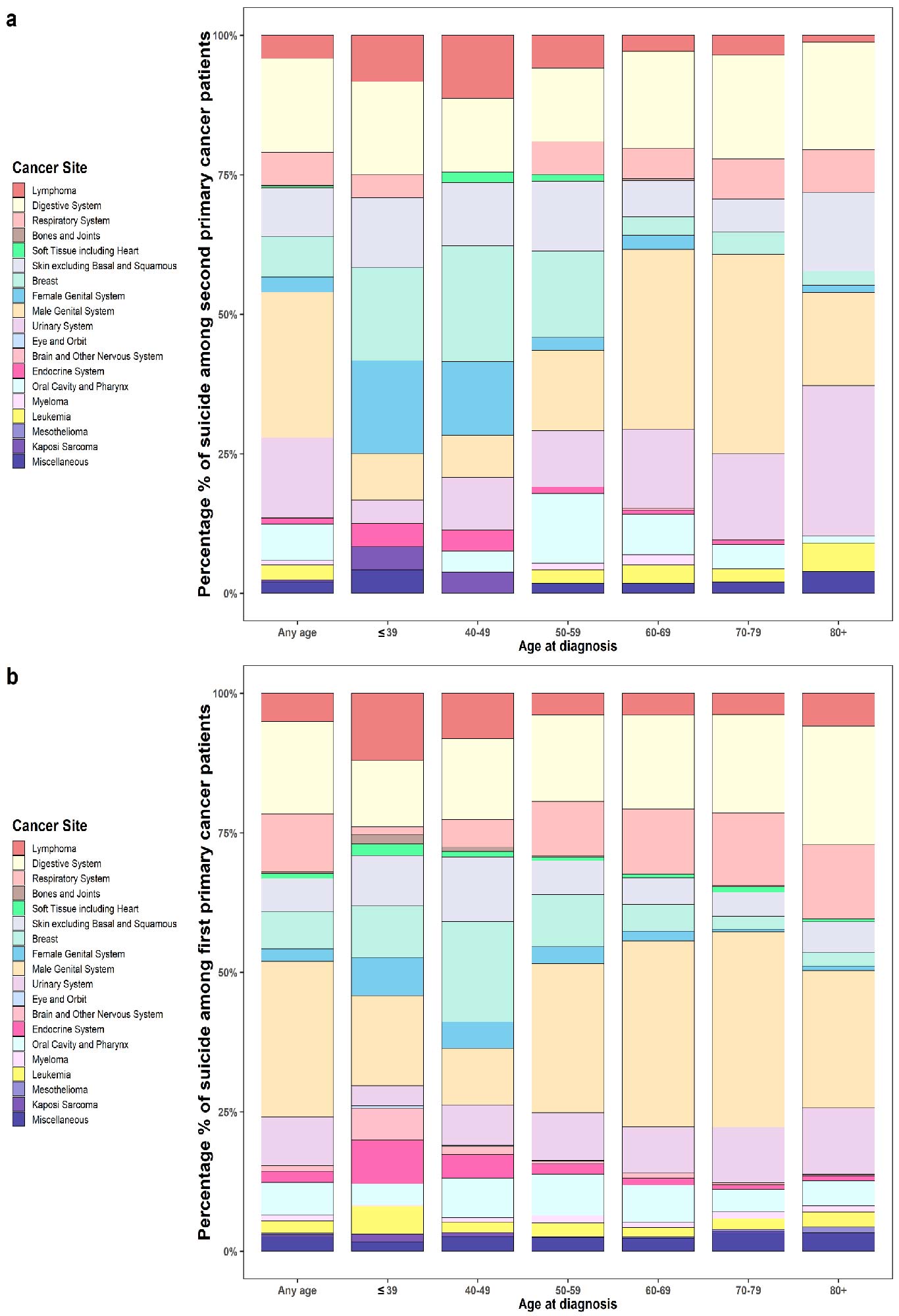
**

**Supplementary Figure 3. Kaplan-Meier survival analysis of patients with first primary cancer**

**
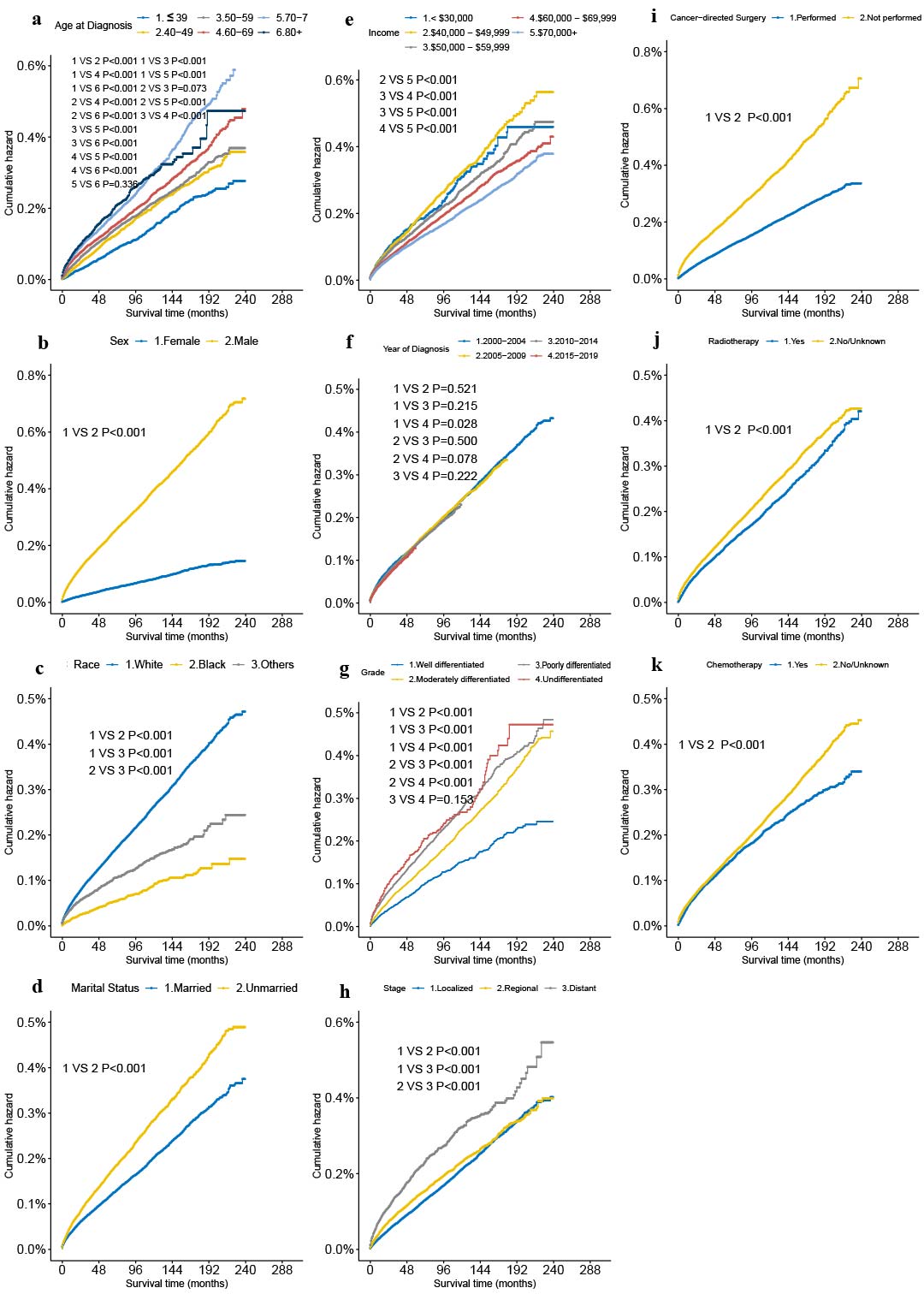
**

**Supplementary Figure 4. Multivariate Cox proportional hazards regression analyses of patients with first primary cancer**

**
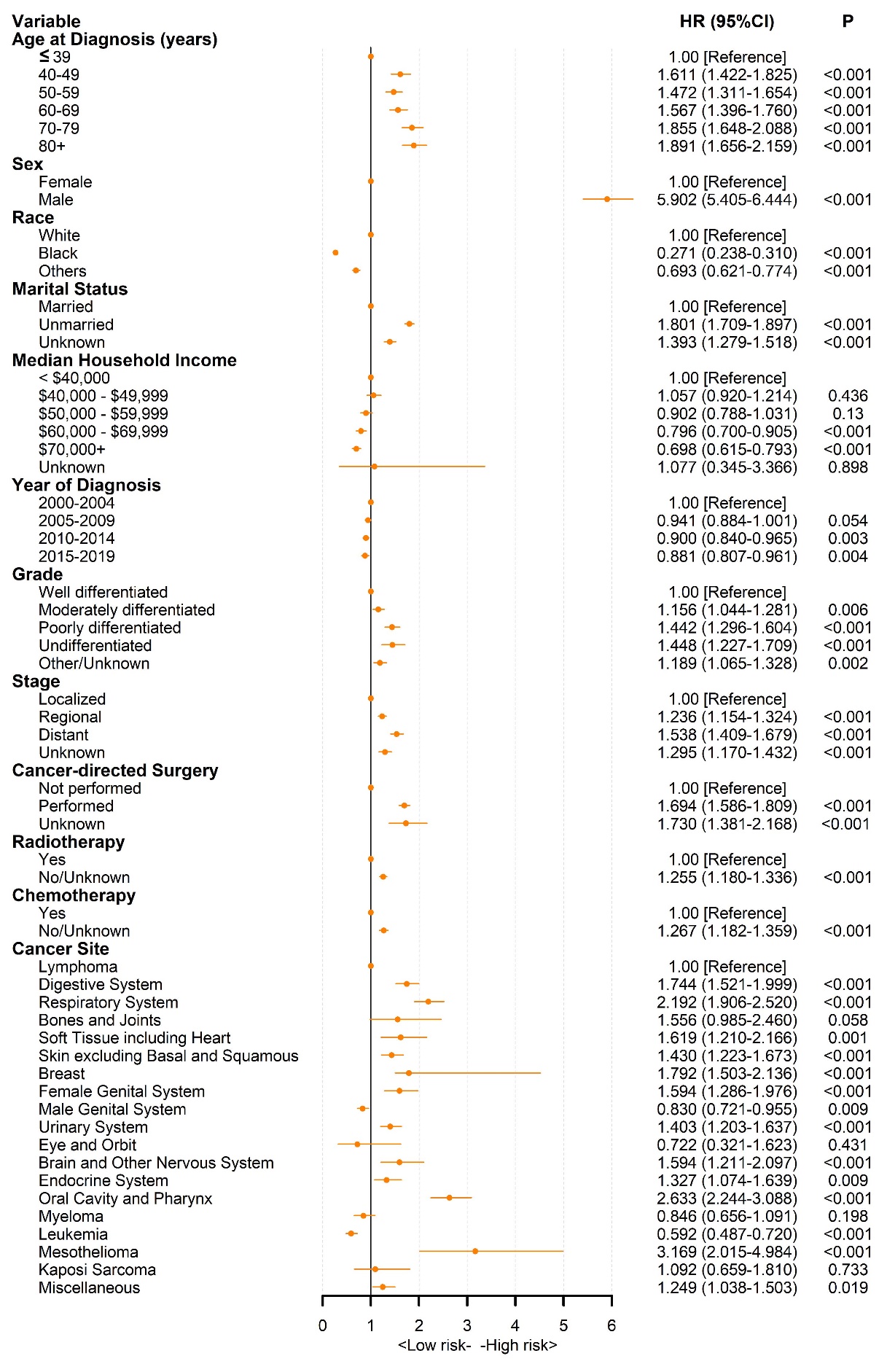
**
